# Diversity and recombination analysis of *Cotton leaf curl Multan virus*: a highly emerging begomovirus in northern India

**DOI:** 10.1186/s12864-019-5640-2

**Published:** 2019-04-06

**Authors:** Razia Qadir, Zainul A. Khan, Dilip Monga, Jawaid A. Khan

**Affiliations:** 1 Plant Virus Laboratory, Department of Biosciences, Jamia Millia Islamia (Central University), New Delhi, 110025 India; 2 Central Institute for Cotton Research (ICAR-CICR), Regional Station, Sirsa, Haryana 125055 India; 3 Present address: Department of Plant Molecular Biology, University of Delhi, South Campus, New Delhi, 110021 India

**Keywords:** *Cotton leaf curl Multan virus*, Cotton leaf curl disease, Betasatellite, Alphasatellite, Recombination

## Abstract

**Background:**

Cotton leaf curl disease (CLCuD), caused by begomoviruses in association with satellite molecules, is a major threat to cotton production causing enormous losses to cotton crop in most of the cotton growing countries including Indian subcontinent. In this study, isolates of begomovirus and satellite molecules associated with CLCuD were collected from North India (Haryana, New Delhi). They were amplified employing rolling circle replication mechanism, cloned, sequenced and, their phylogenetic and recombination analysis was performed.

**Results:**

The five *Cotton leaf curl Multan virus* (CLCuMuV) isolates investigated in this study showed monopartite organization of the genome typical of Old World begomoviruses. Nucleotide sequence analyses assigned them as the strains of CLCuMuV and were designated as CLCuMuV-SR13, CLCuMuV-SR14, CLCuMuV-ND14, CLCuMuV-ND15 and CLCuMuV-SR15. The genome of CLCuMuV-SR13 shared a highest level of nucleotide sequence identity (98%) with CLCuMuV (JN678804), CLCuMuV-SR14 and CLCuMuV-SR15 exhibited 96% with CLCuMuV (KM096471), while isolates CLCuMuV-ND15 and CLCuMuV-SR15 revealed 96% sequence identity with CLCuMuV (AY765253). The four betasatellite molecules investigated in this study shared 95–99% nucleotide sequence identity with *Cotton leaf curl Multan betasatellite* (CLCuMB) from India. The betasatellite molecules were designated as CLCuMB-SR13, CLCuMB-SR14, CLCuMB-ND14 and CLCuMB-ND15. Alphasatellite molecules in this study, designated as GLCuA-SR14, GLCuA-ND14 and GLCuA-SR15, revealed 98% identity with *Guar leaf curl alphasatellite* (GLCuA) reported from Pakistan.

**Conclusion:**

The phylogenetic and recombination studies concluded that the isolates of CLCuMuV genomes undertaken in this study have a potential recombinant origin. Remarkably, significant recombination was detected in almost all the genes with contribution of *Cotton leaf curl Kokhran Virus* (CLCuKoV) in IR, *V1, V2, C1, C4* and *C5* regions and of CLCuMuV in *C2* region of CLCuMuV-SR14. CLCuKoV also donated in *C2, C3* regions of CLCuMuV-ND14; *V1, V2, C2 and C3* regions of CLCuMuV-ND15 and *C1* of CLCuMuV-SR15. Altogether, these observations signify the uniqueness in Indian CLCuMuV isolates showing contribution of CLCuKoV in all the genes. An interesting observation was frequent identification of GLCuA in CLCuD leaf samples.

**Electronic supplementary material:**

The online version of this article (10.1186/s12864-019-5640-2) contains supplementary material, which is available to authorized users.

## Background

India is the largest cotton producing country in the world [1]. Cotton (*Gossypium hirsutum*, family *Malvaceae*) is a shrub cultivated in tropical and subtropical countries for its soft, fluffy and staple fiber. In Mexico, wild cotton species show greatest diversity followed by Australia and Africa. *G. hirsutum*, an important cash crop, is prone to infection by several pathogens and insects. Among them begomoviruses associated with cotton leaf curl disease (CLCuD) cause severe losses to cotton crop [2–4].

CLCuD is induced by complexes of cotton infecting begomoviruses (family *Geminiviridae*) intimately associated with satellite molecules viz. betasatellite and alphasatellite. The family *Geminiviridae* comprises of viruses having single-stranded (ss), circular DNA genomes which are encapsidated in geminate particles [5, 6]. On the basis of genome organization, nucleotide sequence similarities, insect vector and host range, geminiviruses are presently grouped into nine genera viz. *Becurtovirus, Begomovirus, Capulavirus, Curtovirus, Eragovirus, Grablovirus, Mastrevirus, Topocovirus* and *Turncurtovirus* [7]. Among them, *Begomovirus*, the largest and most important genus of the family *Geminivirideae*, presently comprises of > 200 species. They are transmitted by *Bemisia tabaci* (whitefly) insect vector and infect dicotyledonous plants [8, 9]. Five species of begomovirus complexes i.e., *Cotton leaf curl Multan virus* (CLCuMuV), *Cotton leaf curl Bangalore virus* (CLCuBaV), *Cotton leaf curl Kokhran virus* (CLCuKoV), *Cotton leaf curl Gezira virus* (CLCuGeV) and *Cotton leaf curl Alabad virus* (CLCuAlV) are presently associated with CLCuD [10]. The CLCuD-affected cotton plants show characteristic symptoms of thickening and darkening of veins, curling of leaves (mostly upward), development of outgrowths (enations) on the lower side of leaves and stunted growth of plants. This disease spreads through whitefly in a persistent, circulative manner [9, 11].

In 1967, CLCuD was reported for the first time in Multan, Pakistan [12]. Later in 1980, it unexpectedly decreased cotton productivity causing great concern to cotton growers and agricultural scientists. In 1992–1997, it again reduced the yield by 29% [13]. In India CLCuD was first reported from Sri Ganganagar in 1993 [14]. Recently, several variants of CLCuKoV-Bu strain have been reported from India and Pakistan, thus posing an alarming situation which may lead to another CLCuD outbreak in the subcontinent [15]. The recombinant origin of begomoviruses significantly contributes to their increasing pathogenicity [16–20].The genome of begomoviruses associated with CLCuD (henceforth referred to as BAC) is monopartite consisting of a circular, single stranded (ss) DNA molecule (~ 2.7 kb). It is organized into 7 ORFs viz. *C1*, *C2*, *C3*, *C4* and *C5* in complementary sense and *V1* and *V2* in virion sense. The ORFs participate in replication (*C1* and *C3*), transcription activation (*C2*) and packaging (*V1* and *V2*). Though the exact function of *C4* and *C5* genes is still not known, C4 protein participates in suppression of RNA silencing mechanisms and C5 protein, which is not common, may be involved in replication of DNA but remains insignificant in viral infection [21]. The opposing complementary sense and virion sense genes are separated by a conserved non-coding intergenic region (IR) which is called as the common region (CR), consisting of about 200 nts having a highly conserved nonanucleotide (TAATATTAC) sequence containing an origin of replication (*ori*) [22].

Monopartite begomoviruses have also been found to be associated with circular, ss satellite molecules-termed as betasatellites are ~ 1.35 kb (approximately half the size of the monopartite begomovirus DNA genome). They are required for induction of disease symptoms in their host plants [23, 24]. The replication, movement and encapsidation of betasatellites relies on helper virus. The function of betasatellites is governed by the βC1 protein, which functions as a pathogenicity determinant [25, 26].

Some begomovirus-betasatellites are also associated with alphasatellites [11]. The size of alphasatellite is nearly half of the begomoviral genome (1.3–1.4 kb). They are able to replicate autonomously. The alphasatellite encodes a replication associated protein (Rep), which depends on helper begomovirus for its movement and whitefly-mediated transmission in plants [27]. Alphasatellite regulates the virulence of begomovirus-betasatellite complex [28, 29]. Another distinct class of non-coding DNA satellite has been found to be associated with some begomoviruses termed as deltasatellite. The size of deltasatellite is about one quarter of the begomovirus genome (~ 700 nt) and its function is still not known [30].

The genome of begomovirus replicates via rolling circle replication mechanism. Geminiviruses can also replicate their dsDNA via recombination-dependent replication mechanism [31–33]. It is well established that recombination plays an important role in the establishment, emergence and evolution of new species of geminiviruses. The exchange of genetic material via recombination causes plant virus evolution [34]. The network of relationships among various begomoviruses in the Indian subcontinent could also be explained through recombination [35].

In this study, complete genome of several isolates of CLCuMuV, associated betasatellites and alphasatellites were cloned and sequenced. Since the biodiversity of begomoviruses in North India is very rich, the recombination events in the viruses were investigated and analysed for their possible role in the evolution of virus. Infectious clones of CLCuMuV as well as CLCuMB were prepared and agroinoculated in *Nicotiana benthamiana* plants. Furthermore, phylogenetic analysis and potential recombination of isolates of CLCuMuV with reference to other begomoviruses were studied to understand the CLCuD-begomovirus complexity and evolution in the Indian subcontinent.

## Results

### Confirmation of BAC complex

The samples taken in this study from Sirsa during the years 2013, 2014 and 2015 were designated as CLCuMuV-SR13, CLCuMuV-SR14, and CLCuMuV-SR15, respectively and from New Delhi during 2014 and 2015 were abbreviated as CLCuMuV-ND14 and CLCuMuV-ND15. PCR- based amplification of the CP gene confirmed the presence of beomoviral genome in CLCuD cotton samples (Additional file 1: Figure S1). Additionally, 1 μg of Rolling Circle Amplification (RCA) product obtained from CLCuD-infected leaves following digestion with restriction enzyme *Kpn*I yielded a DNA fragment (~ 2.7 kb), thus confirming the presence of the begomoviral DNA in CLCuD-symptomatic leaf samples.

### Nucleotide sequence analysis of genome of BAC and its relationship with other begomoviruses

PCR amplification using RCA product as the template yielded the amplicons of expected sizes (~ 1.2 kb employing primers F1For/F1Rev, and ~ 1.7 kb with primers F2For/F2Rev). The complete nucleotide sequences of five different BAC shared 96–98% nucleotide sequence identity with CLCuMuV, thus were regarded as the isolates of CLCuMuV based on recent species demarcation threshold of begomovirus [36]. Hence, these isolates having genomes of 2757, 2748, 2751, 2751 and 2750 nts were designated as CLCuMuV-SR13, CLCuMuV-SR14, CLCuMuV-ND14, CLCuMuV-ND15 and CLCuMuV-SR15, respectively. Their DNA sequences were submitted to GenBank database with accession numbers KJ868820, KX951460, KX951461, KY561820 and KY888163, respectively. The genome of CLCuMuV-SR13 (KJ868820) isolate shared the highest identity (98%) with CLCuMuV (JN678804).while isolates CLCuMuV-SR14 (KX951460) and CLCuMuV-SR15 (KY888163) exhibited highest identity (96%) with CLCuMuV (KM096471) while CLCuMuV-ND14 (KX951461) and CLCuMuV-ND15 (KY561820) isolates shared highest identity (96%) with CLCuMuV (AY765253). Nucleotide sequences of all the isolates displayed typical begomoviral features with respect to size, organization and ORFs arrangement of the genome. Isolates from New Delhi (ND14 and ND15) possess genome of the same size (2751 nts), while in Sirsa isolates (SR14 and SR15) it was 2748 and 2750 nts, respectively. The difference of three nts was observed within the *Rep* region. The Sirsa isolate (SR13) showed a difference of 9–12 nts in the *V2* region, while the lengths of *V1, C2, C3* and *C4* were similar in all the isolates of this study, a difference of 3–12 nts was observed in *V2* and *Rep* regions (Additional file 2: Table S1).

**Fig. 1:**
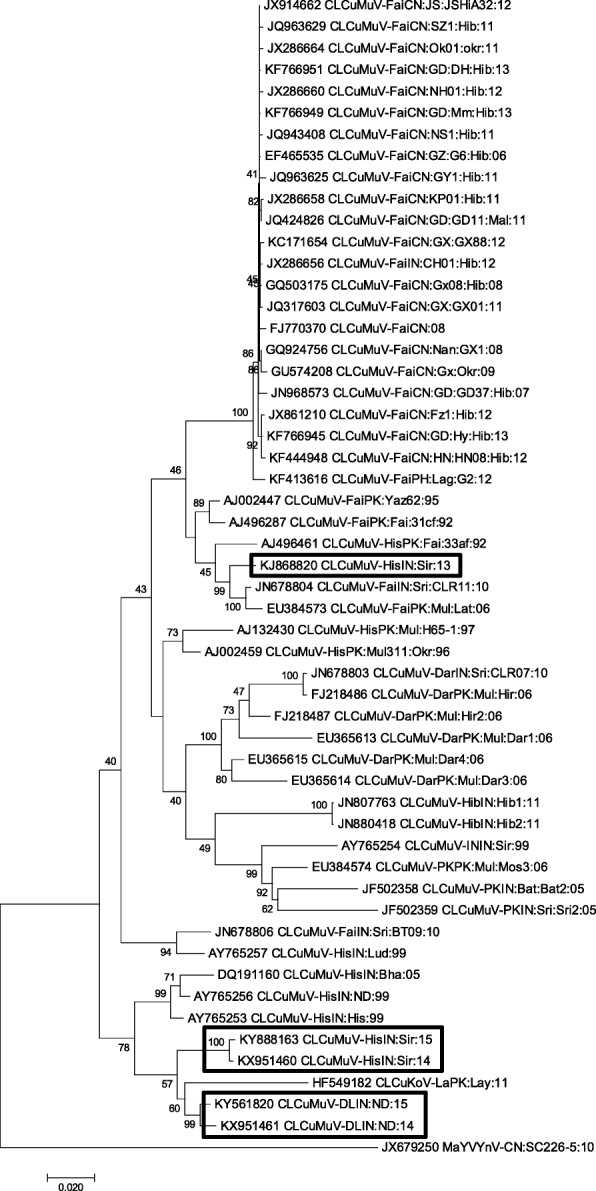
Phylogenetic relationship of full-length genome sequences of *Cotton leaf curl Multan virus* (CLCuMuV) isolates under study (highlighted) with other closely-related members of begomoviruses. The evolutionary distances were obtained by applying Maximum Composite Likelihood approach, and the evolutionary history was estimated using the Neighbor-Joining method in MEGA7. Bootstrap score percentage of 1000 replicates are indicated at each node

Dendrograms were drawn to analyse the phylogenetic relationship of the CLCuMuV isolates from the present study which formed clusters and showed a close relationship with the CLCuMuV isolates from different regions of India and Pakistan. Sequences, derived from Sirsa (SR14-KX951460 and SR-15KY888163) and New Delhi isolates (ND14-KX951461 and ND15-KY561820) along with previously reported CLCuKoV sequences (HF549182) from Pakistan, formed a new clade with CLCuMuV sequences (AY765253, AY765256 and DQ191160) from India; that further diverged into 2 subclades differentiating New Delhi isolates from that of Sirsa isolates, thus signifying their divergence from a common progenitor. However, sequence KJ868820 (SR13) obtained from the Sirsa samples collected from the same site (in the year 2013) clustered separately with CLCuMuV sequences from Pakistan (EU384573, AJ496461, AJ496287 and AJ002447) and India (single isolate JN678804) (Fig. 1). Hence, it shows emergence and variability of CLCuMuV sequences in the Indian subcontinent.

The heat map representing the pairwise sequence alignment generated by Sequence Demarcation Tool version 1.2 (SDT, 58) showed that the genome of CLCuMuV isolates shared more than 91% of sequence identity with other relevant BAC sequences used in this analysis (Additional file 3: Figure S2 a-e) which is more than the cutoff value (91%) for demarcation of begomovirus species [36].

The amplification and sequencing of full-length (~ 2.7 kb) genome obtained using oligo primers (viz. Cot For./Cot Rev.) further confirmed the presence of CLCuMuV in all the RCA derived samples used in the present study.

### Sequence comparison and phylogenetic analysis of satellite molecules

**Fig. 2:**
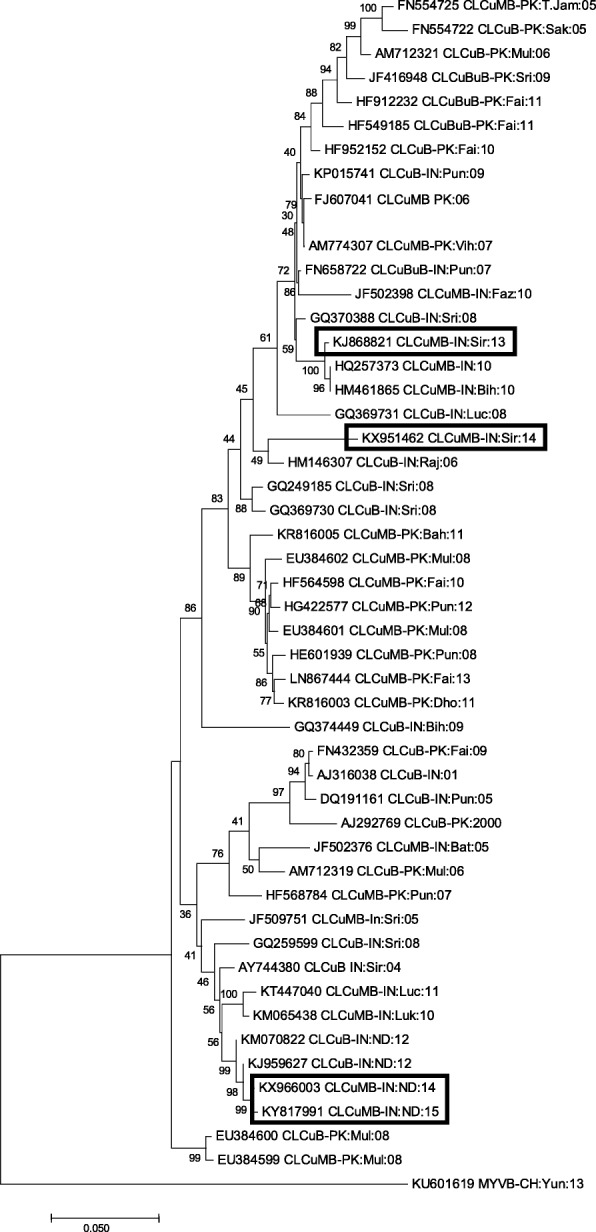
Phylogenetic relationship of *Cotton leaf curl Multan betasatellite* (CLCuMB) under study (highlighted) with other closely-related betasatellite molecules. The evolutionary distances were obtained by applying Maximum Composite Likelihood approach, and the evolutionary history was estimated using the Neighbor-Joining method in MEGA7. Bootstrap score percentage of 1000 replicates are indicated at each node

Employing primers specific to both betasatellite and alphasatellite molecules using RCA product from the CLCuD leaves as the template, PCR amplicons of expected sizes of the betasatellite and alphasatellite were obtained. The nucleotide sequences of betasatellite molecules under study shared 95 to 99% identity with CLCuMB sequences, and were designated as CLCuMB-SR13 (KJ868821), CLCuMB-SR14 (KX951462), CLCuMB-ND14 (KX966003) and CLCuMB-ND15 (KY817991). These sequences possess characteristics features of CLCuMB with respect to typical genome size, organization having single ORF in the complementary sense, SCR (conserved among all betasatellites) and a sequence rich in adenine (A-rich region). The sequences of CLCuMB molecules from New Delhi (KX966003-ND14 and KY817991-ND15) were of the same size (1371 nts), while those from Sirsa (KJ868821-SR13 and KX951462-SR14) were of 1347 and 1362 nts, respectively. Interestingly, the size of β*C1* gene (357 nts) was similar in CLCuMB-SR14 (KX951462), CLCuMB-ND14 (KX966003) and CLCuMB-ND15 (KY817991) but differed in CLCuMB-SR13, KJ868821) due to the presence of an extra 6 nts in sequence (Additional file 4: Table S2). The betasatellite sequence of CLCuMB-SR13 (KJ868821) revealed a maximum identity of 99% with CLCuMB (HQ257373) from India followed by other sequences (HM461865, GQ370388,..etc). CLCuMB-SR14 (KX951462) shared the highest identity (95%) with sequence HM146307, whereas CLCuMB-ND14 (KX966003) and CLCuMB-ND15 (KY817991) exhibited the highest identity (99%) with recently isolated CLCuMB (KJ959627) from New Delhi followed by other sequences, such as, KM070822, KM065438, KT447040…etc., obtained from North India during 2010–2012 [37]. The phylogenetic analysis of CLCuMB sequence studied here formed close clusters with CLCuMB sequences derived from different regions of India. The sequences from Sirsa [CLCuMB-SR13 (KJ868821), CLCuMB-SR14 (KX951462)] formed a group with those reported from northwestern India (HQ257373, HM461875, GQ370388 and HM146307), while CLCuMB sequences from New Delhi [CLCuMB-ND14 (KX966003) and CLCuMB-ND15 KY817991)] clustered with those of North India (KM070822, KJ959627, KM065438 and KT447040). Therefore, based on nucleotide sequence identity, newly identified CLCuMB sequences from two geographical locations in India appear to fall in two different clades in the phylogenetic tree. (Fig. 2).

**Fig. 3:**
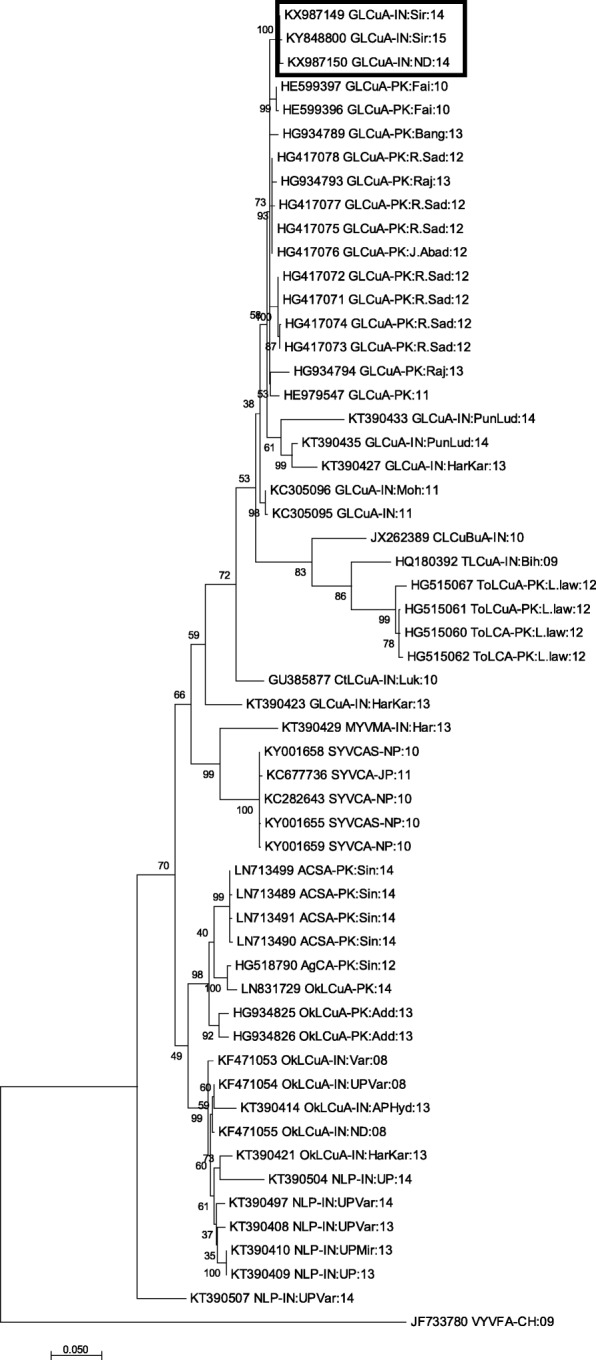
Phylogenetic relationship of *Guar leaf curl alphasatellite* (GLCuA) under study (highlighted) with other closely-related alphasatellite molecules. The evolutionary distances were obtained by applying Maximum Composite Likelihood approach, and the evolutionary history was estimated based Neighbor-Joining method in MEGA7. Bootstrap score percentage of 1000 replicates are indicated at each node

The alphasatellite isolates reported here shared 98% identity with GLCuA and were designated as GLCuA-SR14 (KX987149, 1368 nts), GLCuA-ND14 (KX987150, 1361 nts) and GLCuA-SR15 (KY848800, 1368 nts). These sequences had a typical genome size and organization of alphasatellite with single Rep gene on the virion-sense and an A-rich region. GLCuA isolates from Sirsa (KX987149 and KY848800) and New Delhi (KX987150) showed a difference of 7 nts in full length alphasatellite genome but a difference of 99 nts was observed in the size of Rep gene (Additional file 5: Table S3). Nucleotide sequence alignment studies demonstrated that GLCuA-SR14 (KX987149) and GLCuA-SR15 (KY848800) shared a maximum sequence identity (98%) with GLCuA (HE599397), whereas GLCuA-ND14 (KX987150) revealed the highest identity (98%) with GLCuA (HG417075). Alphasatellites investigated in this study formed a cluster and showed close relationship with GLCuA sequences (HE599397, HE599396, HG934789, HG417078, HG934793, HG417077, HG417075, HG417076,…etc) reported from Pakistan during 2010–2013 (Fig. 3).

SDT analysis showed that in the present study CLCuMB shared > 90% identity with other relevant betasatellites used in SDT analysis (Additional file 6: Figure S3 a-d), while alphasatellites displayed > 92% identity with other alphasatellite sequences used in the analysis (Additional file 7: Figure S4 a-c).

### Recombination analysis

Since recombination is the leading aspect in the evolution and emergence of geminiviruses [14, 38], we investigated the evidence of recombination in the nucleotide sequences of newly identified CLCuMuV isolates and associated satellites using RDP4 (Recombination detection program). The analysis was performed by comparing the present sequences of BAC with various sequences of begomoviruses retrieved from GenBank database. It displayed potential recombination events with distinct patterns in several full-length genomes of newly- identified CLCuMuV and associated betasatelllite and alphasatellite sequences as listed below:

#### Cotton leaf curl Multan virus

**Fig. 4:**
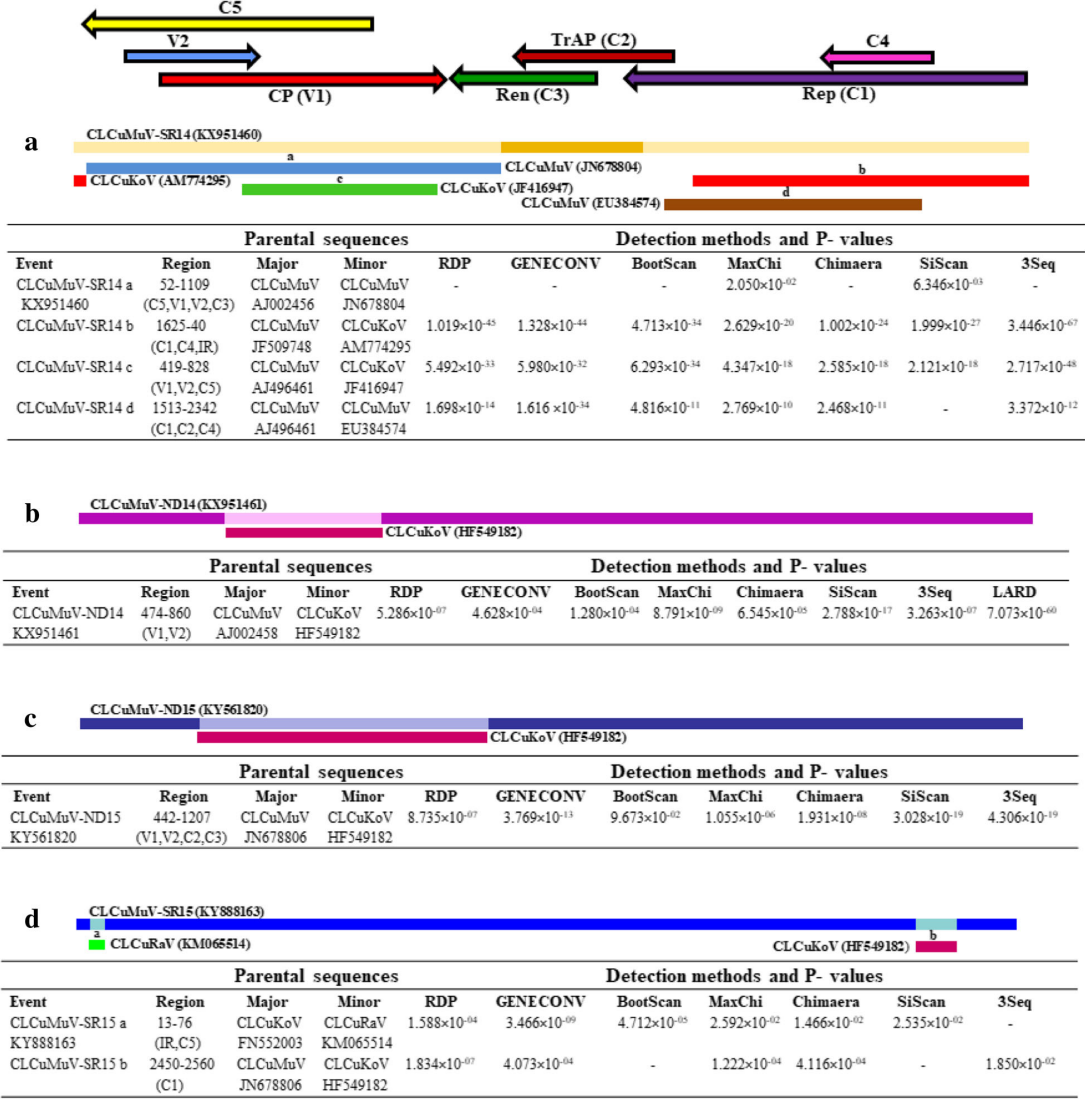
**(a**-**d)** Recombination events observed in full-length nucleotide sequences of *Cotton leaf curl Multan virus* (CLCuMuV) of the present study using RDP4. Genome map of CLCuV is displayed at the top. Different coloured bars representing different recombinant fragments along with their minor parents are shown. Detailed features of each recombinant fragment viz. putative parental sequences, recombination region and the algorithms with average P-values generated in this analysis are also listed

Significant recombination events were observed at four sites in CLCuMuV-SR14, involving CLCuMuV (JN678804), CLCuKoV (AM774295), CLCuKoV (JF416947), and CLCuMuV (EU384574) respectively, as minor parents (Fig. 4a). Except for the recombination event contributed by CLCuMuV (JN678804), all the events were considered likely as they were supported by more than 3 methods of RDP with significant *P*-values. The contribution of CLCuKoV (AM774295) in the recombinant fragment (nt positions at 1625–2748 and 1–40) involved IR, *C1, C4* and regions, while from CLCuKoV (JF416947), it involved *V1*, *V2* and *C5* regions in fragment 419–828 (Fig. 4a). CLCuMuV (EU384574) showed contribution in *C1, C2* and *C4* regions involving nt position 1513–2342. Except *C3* region, the significant recombination events were detected in all the genes (*V1, V2, C1, C2, C4* and *C5*) of CLCuMuV-SR14 (KX951460).

In isolates CLCuMuV-ND14(KX951461) and CLCuMuV-ND15 (KY561820), recombinant fragments signified CLCuKoV (HF549182) as a common parental sequence and differed only in length of their spanning regions. Interestingly, CLCuKoV (HF549182) contributed in the *V1* and *V2* regions of CLCuMuV-ND14 (KX951461) at nt position 474–860, and in *V1, V2, C2* and *C3* regions of CLCuMuV-SR14 (KX951460) at nt position 442–1207 (Fig. 4b, c).

In CLCuMuV-SR15 (KY888163), two different recombination events were detected depicting *Cotton leaf curl Rajasthan Virus* (CLCuRaV, KM065514) and CLCuKoV (HF549182) as minor parents (Fig. 4d). CLCuKoV (HF549182) contributed in *C1* region (nt position 2450–2560), and a recombinant fragment of 13–76 nts involving IR and *C5* was donated by CLCuRaV (KM05514) in the genome of CLCuMuV- SR15 (KY888163). Recombination events detected in CLCuMuV-ND14, CLCuMuV-ND15 and CLCuMuV-SR15 were supported by more than 3 methods in RDP.

Nucleotide sequences analysed in this study, including putative parental sequences of these isolates have shown significant recombination breakpoints in IR*, V1, C1* and *C2* regions which further supports the previous studies [34, 37, 39]. The RDP analysis has depicted that the putative parents of the present virus isolates, and also other viruses included during analysis, have evolved via recombination at some point of time. Thus, suggesting that the mechanism of recombination greatly affects the begomoviral evolution.

#### Cotton leaf curl Multan betasatellite

**Fig. 5:**
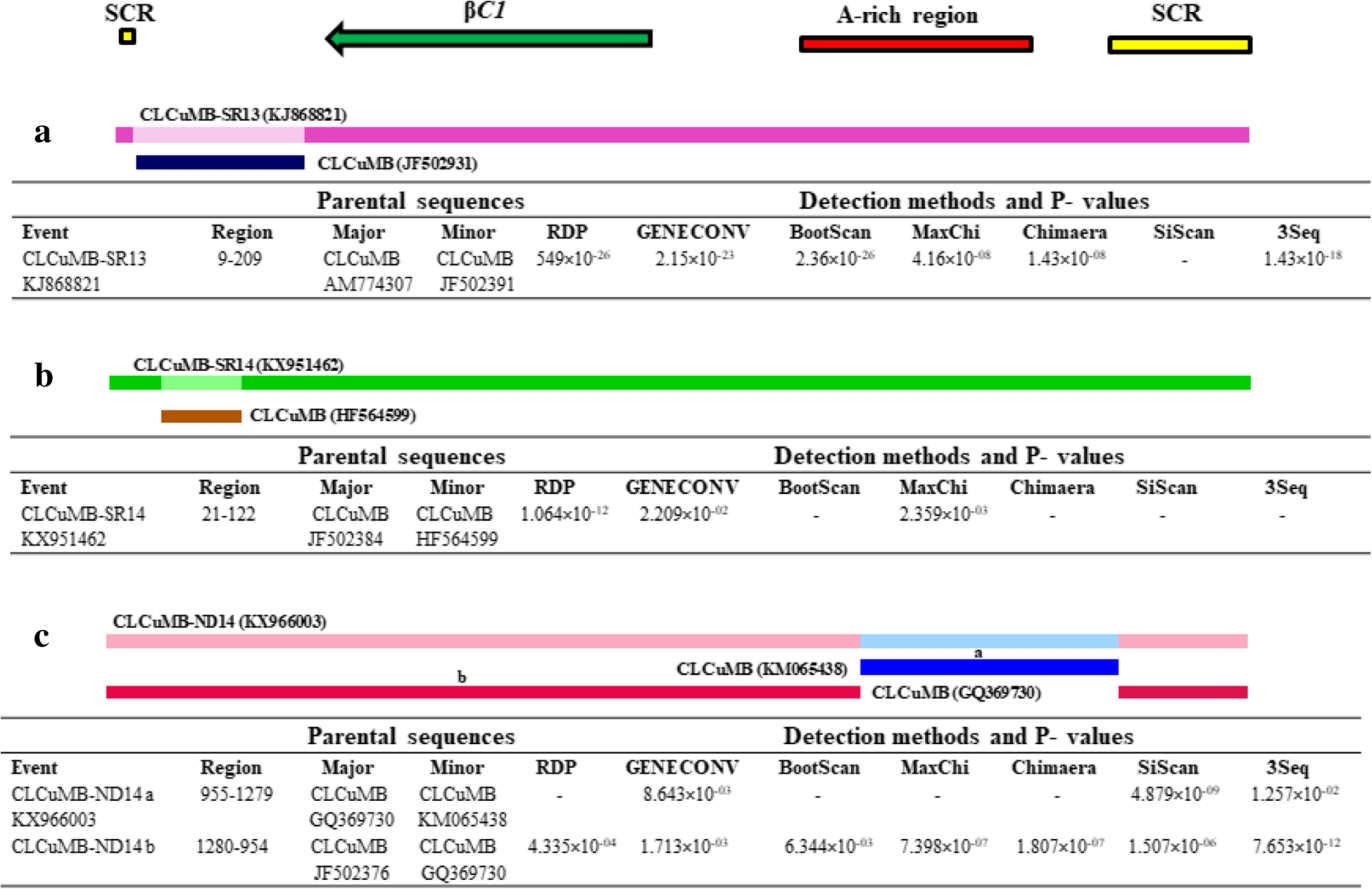
**(a**-**c)** Recombination events detected in *Cotton leaf curl Multan betasatellite* (CLCuMB). Genetic map of betasatellite is shown at the top. Different coloured bars representing different recombinant fragments along with their minor parents are shown. Detailed features of each recombinant fragment viz. putative parental sequences, recombination region and the algorithms with average P-values generated in this analysis are also listed

In CLCuMB-SR13 (KJ868821), a recombinant portion was detected at nt position 9–209 which includes SCR and a short stretch of C-terminal of β*C1* gene with CLCuMB (JF502391) as minor parent and CLCuMB (AM774307) as major parent (Fig. 5a). Similarly, in CLCuMB-SR14 (KX951462), recombination event was detected in satellite conserved region (SCR) at position 21–122 with CLCuMB (HF564599) as minor parent and CLCuMB (JF502384) as major parent (Fig. 5b).

CLCuMB-ND14 (KX966003) displayed recombination events at two sites. One at nt position 955–1279 involving SCR and A-rich regions with CLCuMB (KM065438) as minor parent and CLCuMB (GQ369730) as major parent. The other recombination site spanned nt position 1280–954 involving SCR, β*C1* and A-rich regions with CLCuMB (GQ369730) as minor parent and CLCuMB (JF502376) as major parent (Fig. 5c).

#### Alphasatellites

**Fig. 6:**
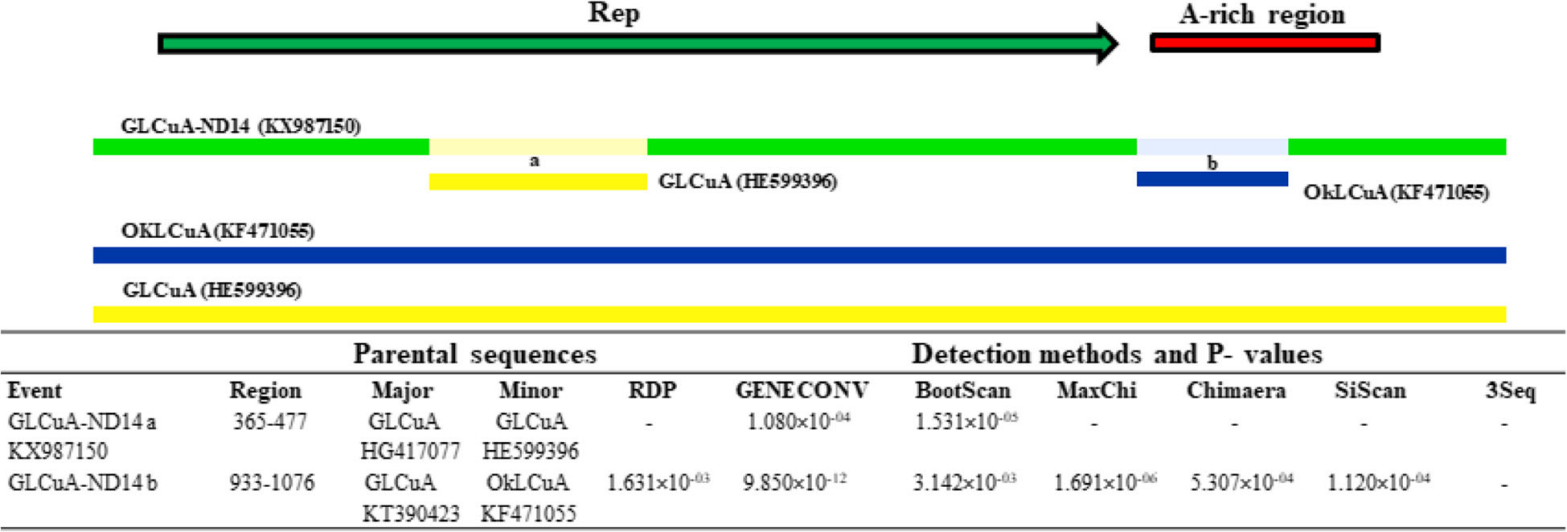
Recombination events detected in nucleotide sequence of *Guar leaf curl alphasatellite* (GLCuA) sequence. Genetic map of GLCuA is presented at the top. Different coloured bars representing different recombinant fragments along with their minor parents are shown. Detailed features of each recombinant fragment viz. putative parental sequences, recombination region and the algorithms with average P-values generated in this analysis are also listed

Among sequences of different alphasatellite molecules, recombination events were detected in GLCuA-ND14 (KX987150) at two sites, one within the Rep region (nt position 365–477) with GLCuA (HE599396) as minor parent and GLCuA (HG417077) as major parent. The other recombination event covered nt position 933–1076 involving Rep and A-rich region with *Okra leaf curl alphasatellite* (OkLCuA, Acc. KF471055) as minor parent and GLCuA (KT390423) as major parent. It was considered likely as the event was detected by > 3 methods (Fig. 6).

### Infectivity of cloned DNA

**Fig. 7:**
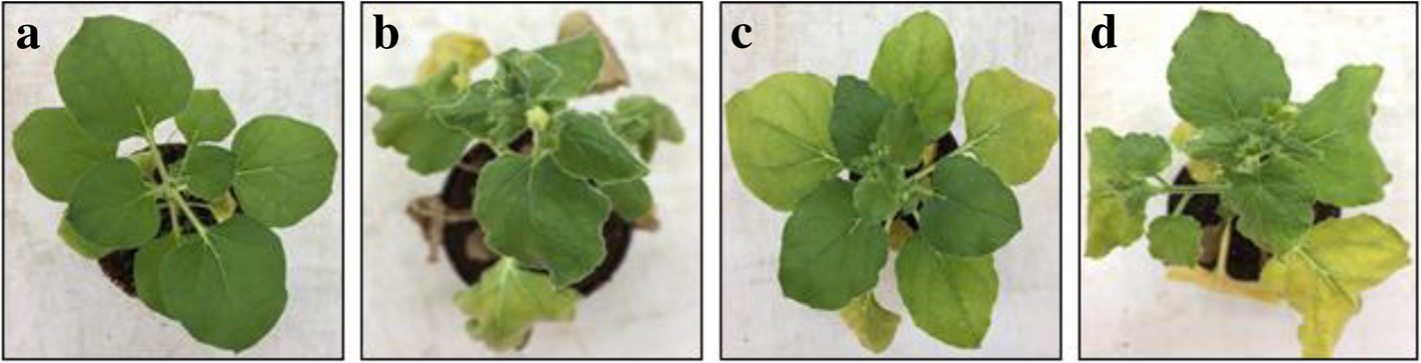
*Nicotiana benthamiana* plants showing symptoms after agroinoculated with mock sample (**a**), infectious clones of the *Cotton leaf curl Multan virus (CLCuMuV)* (**b**), *Cotton leaf curl Multan betasatellite* (CLCuMB) (**c**) and both CLCuMuV and CLCuMB (**d**)

Infectious clone of CLCuMuV (pGR5.4) upon agroinfiltration in leaves of *N. benthamiana*, developed mild symptoms. However, in combination with betasatellite (pCAMβ), symptoms such as vein thickening, yellowing and curling of leaves were observed at 14 days post infiltration in *N. benthamiana* (Table 1; Fig. 7). Thus, it demonstrated that CLCuMuV is a betasatellite-dependent begomovirus for symptom induction. The CLCuMuV, when alone, could infect *N. benthamiana*, turning yellowing of leaves. However, additional betasatellite was required for induction of leaf curl symptoms.

**Table 1:** The infectivity analysis of *Cotton leaf curl Multan virus* and *Cotton leaf curl Multan betasatellite* in *Nicotiana benthamiana* plants

Inoculums	Plant infected/Plant inoculated (*Nicotiana benthamiana*)	Days to infection	Symptoms
pGR5.4	8/15	14	Downwards leaf curling, and vein thickening
pCAMβ	0/15	–	No symptoms
pGR5.4 + pCAMβ	11/15	14	Downwards leaf curling, leaf swelling, vein thickening and stunting
Mock (pGR5.4 *in E. coli*)	0/15	–	No symptoms

## Discussion

The present study was taken up as it deemed desirable to understand the complexity of this disease. Cotton leaves showing CLCuD symptoms were taken from Haryana and New Delhi, India. RCA was performed for characterization and phylogenetic analysis of begomoviruses,, betasatellite and alphasatellite molecules associated with CLCuD from North India. Nucleotide sequences of all the BAC showed the typical size and organization of typical monopartite begomovirus [40–43]. The isolates of the BAC in the present study exhibited a maximum nucleotide sequence identity with CLCuMuV sequences (JN678804, AY765253 and DQ191160) reported earlier from different parts of India [27]. The sequence similarity was further supported by phylogenetic analysis. The clustering of the begomoviral isolates with CLCuMuV is consistent with nucleotide sequence alignments. Further, phylogenetic studies demonstrated that isolates from the same region (Sirsa, Haryana) collected in different years fell under two separate groups. The formation of separate clusters might be due to difference in the length of *V2* region of CLCuMuV-SR13 as compared to other isolates identified in this study. Also, the nucleotide sequence of CLCuMuV-SR13 (KJ868820) showed highest similarity with CLCuMuV sequences from Pakistan (EU384573, AJ496461, AJ496287 and AJ002447), thus formed a group with Pakistani isolates. Other four newly identified sequenes of CLCuMuV-SR14 (KX951460), CLCuMuV-ND14 (KX951461), CLCuMuV-ND15 (KY561820) and CLCuMuV-SR15 (KY888163) shared maximum identity with CLCuMuV sequences from India (AY765253, AY765256 and DQ191160) and forming a group with Indian isolates. The CLCuMuV isolates in this study also exhibited highest sequence identity amongst themselves indicating the presence of same strain according to recent begomoviral demarcation threshold [36]. This further demonstrates that CLCuD in northern Indian regions is predominantly induced by CLCuMuV [44]. Interestingly, despite high sequence similarity, there exists diversity among genome organization of these isolates. For instance, the genomes of CLCuMuV-SR14 and CLCuMuV-SR15 possess 7 ORFs, whereas those of CLCuMuV-SR13, CLCuMuV-ND14 and CLCuMuV-ND15 contain 6 ORFs. Remarkably, *C5* gene rarely reported in CLCuMuV genome, was identified in CLCuMuV-SR14 and CLCuMuV-SR15.

Betasatellite sequences analysed in this study showed typical genome organization having a single ORF on complementary-sense strand, SCR (conserved amongst all the betasatellites) and a sequence rich in adenine (A-rich region) [40, 45]. These sequences shared a maximum identity with CLCuMB isolates from India and were supported by their clustering pattern in phylogenetic analysis. However, CLCuMB sequences, obtained from two different regions (Sirsa and New Delhi) appeared to form separate clusters. The clustering pattern of CLCuMB sequences from Sirsa seemed to have same ancestral origin but have probably evolved separately. These results depicted the monophyletic nature of CLCuMB which is consistent with the previously reported (pre-2013) CLCuMB sequences from India and Pakistan, contrasting the recently reported (post-2013) CLCuMB sequences from India and Pakistan that form groups regardless of their geographical origin [37, 44, 46].

Alphasatellite sequences obtained in this study also showed a typical pattern consisting of a single virion sense Rep gene and an A-rich region [40]. Notably, none of the alphasatellite reported in the current study shared significant sequence similarity with cotton leaf curl alphasatellite, rather they exhibited a maximum similarity to GLCuA (HE599397, HE599396, HG934789,…etc) from Pakistan. This encouraged us to conclude that the alphasatellite associated with CLCuD isolates of the present study is actually GLCuA. The frequent identification of GLCuA in the present study from CLCuD-infected cotton plants, however, contrasts with the recent detection of CLCuD associated alphasatellites (ToLCA and OkLCuA) by Datta et al., 2017 [44]. Thus, our results revealed a mixed infection and different natural combinations of CLCuMuV and its associated satellites CLCuMB and GLCuA in cotton plants from India. Phylogenetic analysis supported these results by placing the GLCuA sequences on a separate branch closely related to GLCuA from Pakistan (HE599397, HE599396, HG934789,…etc). The occurrence of GLCuA might be due to migration of whitefly vector from the neighboring country Pakistan as suggested by Sahu et al., 2018 [38].

The identification of CLCuMuV in CLCuD cotton plants in North India shows a correlation with the recent literature which documents the rebound of CLCuMuV in Punjab (India) and Vehari in Pakistan [44, 46]. Thus, our observations from North India together with the recent studies from the Punjab regions of India and Pakistan, suggest the recurrence of CLCuMuV in the Indian subcontinent and shows its predominance and frequent emergence in North Indian regions which might cause shift in previous epedemics of the recombination-prone CLCuKoV to this subcontinent [47].

Various factors such as mixed infections, high level of viral replication and increased vector host range could contribute significantly to recombination [48]. The evolution and biodiversity of begomoviruses and also other viruses is mainly attributed to recombination [17]. Our analysis revealed a potential recombination origin of CLCuMuV isolates studied in this article. In previous studies IR*, V1, V2* and *C1* regions were shown to be the recombination hotspots in begomovirus genome [24, 34, 36, 39, 46, 49–51]. In the present study, recombination events were however identified in all the CLCuMuV genes and correlate well with the recent analysis [44]. In spite of similarities, earlier findings differ from this study in several aspects [44]. For instance, recombinant fragments of CLCuMuV were contributed by CLCuKoV and CLCuAlV (as major parents) and CLCuMuV (as minor parent). Inaddition, almost all of the recombinant fragments of CLCuMuV were contributed by CLCuMuV (as major parent) and CLCuKoV (as minor parent) and no evidence of recombination from CLCuAlV was observed in the present study. Further, in previous study CLCuKoV was shown to contribute only the *Rep*, CR and *ori* to CLCuMuV [10]. However, in the present study CLCuKoV showed contribution in genes of both complementary sense and virion sense of the CLCuMuV genome. For instance, in CLCuMuV-SR14, CLCuKoV devoted the IR, *C1*, *C4*, *C5*, *V1* and *V2* regions. Inspite of that, *C1*, *C4, V1, V2* and *C5* regions of this isolate (SR14) were also shared by CLCuMuV. Further, *C3* and *C2* regions were contributed by isolates CLCuMuV (JN678804) and CLCuMuV (EU384574), respectively. In CLCuMuV-ND14, CLCuKoV donated only in the *V1* and *V2* regions and not the *C1Rep*. In CLCuMuV-ND15, CLCuKoV contributed the *V1*, *V2*, *C2* and *C3* genes. Though CLCuMuV isolates from Pakistan having short stretches of *C1*, *C2* and *C3* were reported to be derived from CLCuKoV, in the Indian isolates contribution of CLCuKoV was previously detected only in the IR and *Rep* regions [10]. These observations suggest that the emergence of several new BAC in north Indian regions occurred because of recombination between two distinct species viz. CLCuMuV and CLCuKoV.

In our study nucleotide sequences of CLCuMB showed recombination in the β*C1*, SCR and A-rich regions, thus supporting the previous reports from Pakistan and India [24, 37, 44, 48]. And, in GLCuA, recombination was detected in Rep and A-rich regions which is compatible with earlier studies suggesting these regions as the hotspots of recombination [52].

## Conclusions

In conclusion, this study revealed that CLCuMuV has been evolving continuously and the evolution of recombinant begomovirus isolates reported here might be derived via exchange of genetic material from related begomoviruses viz., CLCuMuV, CLCuKoV and CLCuRaV. Our results clearly demonstrated the complex pattern of inter-species and intra-species recombination leading to significant structural changes in DNA components of CLCuMuV. It thus resulted in the genetic variability and emergence of new variants. This genetic diversification of CLCuMuV may lead to diverse host range (enhancing threat to other crops), transmission ability and capability of breaking resistance, hence posing a big challenge for disease management. This study encourages further analysis about molecular epidemiology and genetic evolution of geminivirus. Timely diagnosis and application of control strategies need to be done before this disease complex spreads over a large scale and causes severe threat to economy. This molecular information may provide insights about the genome complexities, sequence identity and genetic variability parameters viz., recombination analysis of CLCuMuV genome, its associated satellite DNAs (CLCuMB and GLCuA) which are indispensable for developing virus-based disease management strategies. This may accelerate the development of CLCuD-resistant plants. Pathogen-derived resistance manipulating CLCuMuV genes has already shown a great potential to generate virus-resistant plants.

## Methods

### Sample collection and detection of begomoviruses associated with CLCuD (BAC)

Leaves were collected from cotton plants exhibiting CLCuD symptoms at the experimental fields of Central Institute for Cotton Research (CICR Regional Station, 29.54°N 75.04°E), Sirsa, Haryana (20 samples) and from JMI (28.56°N 77.28°E), New Delhi (5 samples) between 2013 and 2015 every year during the months of October. The total genomic DNA from the diseased leaves was isolated employing DNAeasy plant mini kit (QIAGEN, Germany). It was subjected to RCA to amplify full-length genomes of BAC with the help of templiphi DNA amplification kit (GE Healthcare, USA).The concentration of DNA was estimated by Biophotometer plus (Eppendorf, Germany). Presence of the BAC was confirmed following PCR-based amplification of the CP gene using RCA product as template employing oligo primers viz. P1 (5′GGGATTTGATTTCAGTAATAAGG 3′) and P2 (5´ GAGCATGTTGTATATGTAGACCA 3´) specific for the CP gene of BAC [14].

### Amplification, cloning and nucleotide sequencing of the genomes of BAC

PCR-based amplification of entire genomes of BAC was performed using RCA product as template employing overlapping begomovirus-specific overlapping primer pairs viz. F1For/F1Rev and F2For/F2Rev [53]. The desired amplicons (1.2 kb and 1.7 kb) from the gels were eluted and purified employing gel extraction Kit (QIAGEN, Germany) and subsequently cloned in PCR cloning vector pGEMT (pGEM-T easy vector systems, Promega, USA). The authenticity of the clones was confirmed following restriction digestion, PCR amplification and sequencing in both directions by automated sequencer (ABI 3730 XL) at Xcelris Genomics (Ahmedabad, India). Sequence data derived from all the clones was assembled and combined to acquire the full- length genome sequence of BAC. Based on sequences obtained from the PCR amplicons of BAC, a primer pair (viz. Cot For. – 5’GGTACCTGAGTACAGGGGTCTATC 3’/Cot Rev. – 5’AAGCTTAATCAAAGTACAGCACAGGG 3′) was designed and employed to amplify and clone full-length genome of CLCuMuV, which were subjected to nucleotide sequence determination. The sequences obtained from the full-length CLCuMuV genome were compared with those of of BAC clones obtained through the overlapping primer pairs in PCR. Furthermore, the primer pair (viz. Cot For/ Cot Rev) was also used for specific detection of full-length BAC in all the samples investigated.

### Cloning and nucleotide sequencing of satellite molecules (betasatellites/alphasatellites) associated with CLCuD

Amplification of satellite molecules was done using RCA product as the template employing oligo primers specific for betasatellite [54] and alphasatellite [55]. The PCR products were resolved on agarose gel, purified with the help of Gel extraction Kit (QIAGEN, Germany) and cloned into pDrive (QIAGEN, Germany) and pGEM-T easy vector (Promega, USA). Nucleotide sequences of the clones representing betasatellite/alphasatellite were determined using automated sequencer (Xcelris Genomics, Ahmedabad, India).

### Sequence comparison and phylogenetic analysis

Nucleotide sequences derived from different clones representing BAC isolates were assembled to construct complete genome. Sequences of the complete BAC genomes and their associated satellite molecules (this study) were compared with those available in NCBI GenBank database following BLASTn (https://blast.ncbi.nlm.nih.gov) program [56]. A total of 54 full-length sequences of genomes of BAC, 44 betasatellite and 53 alphasatellite sequences originating from the Indian subcontinent and depicting high homology with the sequences determined under study, were selected and retrieved from GenBank (details of sequences used in this analysis are provided in Additional file 8: Table S4; Additional file 9: Table S5; Additional file 10: Table S6). Phylogenetic analysis following multiple sequence alignments was performed using MEGA-7.0 [57]. Default parameter of one character-based and two distance-based algorithms were included. A consensus dendrogram was finally drawn for these algorithms employing bootstrap value of 1000 replicates. Pairwise sequence alignment was performed using SDT [58].

### Recombination analysis

To investigate the extent of recombination, putative breakpoints and parents of CLCuMuV isolates, RDP-4 was employed [59]. The full-length sequences of BAC, betasatellite and alphasatellite were used for recombination analysis (Additional file 11: Table S7 a-c). Nucleotide sequences were aligned using CLUSTAL-W in MEGA 7 [57]. The resultant alignments were exported to the RDP4 and subjected to recombination analysis. Recombination analysis having cutoff of 0.05 was used as a P-value and the default settings was performed in RDP4 programme employing RDP, BOOTSCAN, GENCONV, SISCAN, MAXCHI, CHIMAERA and 3SEQ to estimate the recombination events. Recombination analysis were considered true, if they were supported by at least 3 methods.

### Development of CLCuMuV/CLCuMB-derived infectious clones and agroinoculation into *Nicotiana benthamiana* plants

Infectious clones of the CLCuMuV genome (isolate CLCuMuV-SR13) and associated CLCuMB (CLCuMB-SR13) were cloned in pGreen0029 (pGR5.4) and pCAMBIA1302 (pCAMβ) binary vectors respectively, in accordance with Pratap et al., 2011 [60]. The *Kpn*I based partially digested RCA product generated a DNA fragment of ~ 2.7 kb which was cloned into pGreen0029 vector (pGR2.7I) following its nucleotide sequence determination (Xcelris Genomics, Ahmedabad, India). The RCA product obtained from CLCuD-infected cotton leaves was subjected to PCR amplification using oligo primers (viz. Cot For/Cot Rev. containing *Kpn*I and *Hind*III restriction sites in Forward and Reverse primers, respectively) as described in above section. PCR amplicon (~ 2.7 kb), thus obtained, was cloned into pGEM-T Easy vector (pGEM-T-2.7), digested with *Kpn*I and *Hind*III restriction enzymes (Fermentas, USA) and cloned in pGreen0029 vector (pGR2.7II). The DNA insert from the pGR2.7I vector was released following digestion with *Kpn*I restriction enzyme and subsequently cloned in binary vector pGR2.7II. The complete head to tail dimer of CLCuMuV genome was cloned in pGreen0029 vector pGR5.4. The integration and orientation of dimeric clone of CLCuMuV genome was verified after restriction digestion with *Nco*I.

Similarly, full-length CLCuMB sequence (CLCuMB-SR13), which was previously cloned in pDrive (pDriveβ) vector, was subjected to restriction digestion by *Eco*RI and *Bgl*II. The digested DNA fragment (~ 500 bp) having hairpin loop, thus generated, was cloned into pCAMBIA1302 binary vector (pCAM0.5β). The complete CLCuMB sequence cloned into pDriveβ was released following digestion with *Eco*RI and cloned into pCAM0.5β vector (pCAMβ). Cloning and orientation of the partial dimer was confirmed by restriction digestion. The infectious clone viz. pGR5.4 (along with helper plasmid pSoup) and binary vector (pCAMβ) were mobilized in *Agrobacterium tumefaciens* strain LBA4404 by freeze thaw method [61]. The mobilization of pGR5.4 and pCAMβ was confirmed by colony PCR employing primers specific for BAC (viz. Cot For/Cot Rev) and betasatellite (β01/β02) respectively, as described in above section. The agroinfiltration in *N. benthamiana* plants was done as described by Khan et al., 2015 [8].

## Additional files

Additional file 1: **Figure S1.** PCR based detection of coat protein gene of begomovirus associated with cotton leaf curl disease (BAC) in symptomatic leaf samples using primers specific for the CP gene of BAC. (DOC 324 kb)

Additional file 2: **Table S1.** Nucleotide positions and coding capacity of predicted genes of isolates of *Cotton leaf curl Multan virus*. (DOC 36 kb)

Additional file 3: **Figure S2.** (a-e) Sequence Demarcation Tool based pairwise sequence comparisons. Colour-coded pairwise identity matrix generated from cotton leaf curl disease associated begomovirus genomes. Each coloured cell represents a percentage identity score between two sequences (one indicated horizontally to the left and the other vertically at the bottom). Sequences identified in this study are indicated in the red box. (DOC 963 kb)

Additional file 4: **Table S2.** Position and coding capacity of predicted gene of *Cotton leaf curl Multan betasatellite* molecules associated with cotton leaf curl diseased cotton plants. (DOC 33 kb)

Additional file 5: **Table S3.** Position and coding capacity of predicted gene for *Guar leaf curl alphasatellite* molecules associated with cotton leaf curl diseased cotton plants. (DOC 32 kb)

Additional file 6: **Figure S3**. (a-d) Sequence Demarcation Tool based pairwise sequence comparisons. Colour-coded pairwise identity matrix generated from betasatellite genomes. Each coloured cell represents a percentage identity score between two sequences (one indicated horizontally to the left and the other vertically at the bottom). Sequences identified in this study are indicated in the red box. (DOC 718 kb)

Additional file 7: **Figure S4. (**a-c) Sequence Demarcation Tool based pairwise sequence comparisons. Colour-coded pairwise identity matrix generated from alphasatellite genomes. Each coloured cell represents a percentage identity score between two sequences (one indicated horizontally to the left and the other vertically at the bottom). Sequences identified in this study are indicated in the red box. (DOC 662 kb)

Additional file 8: **Table S4.** List of nucleotide sequences of begomoviruses assocated with CLCuD used for the phylogenetic and SDT analysis. (DOC 73 kb)

Additional file 9: **Table S5.** List of nucleotide sequences of betasatellites used for phylogenetic and SDT analysis. (DOC 67 kb)

Additional file 10: **Table S6.** List of alphasatellite sequences used for phylogenetic and SDT analysis. (DOC 77 kb)

Additional file 11: **Table S7.** List of nucleotide sequences of begomoviruses (a), betasatellites (b) and alphasatellites (c) associated with CLCuD used for recombination analysis. (DOC 39 kb)

## Abbreviations

BAC: Begomoviruses associated with cotton leaf curl disease
CLCuAlV: *Cotton leaf curl Alabad virus*
CLCuBaV: *Cotton leaf curl Bangalore virus*
CLCuD: Cotton leaf curl disease
CLCuGeV: *Cotton leaf curl Gezira virus*
CLCuKoV: *Cotton leaf curl Kokhran Virus*
CLCuMB: *Cotton leaf curl Multan betasatellite*
CLCuMuV: *Cotton leaf curl Multan Virus*
CLCuRaV: *Cotton leaf curl Rajasthan virus.*
CP: Coat Protein
CR: Common Region
GLCuA: *Guar leaf curl alphasatellite*
IR: Intergenic Region
ORF: Open Reading Frame
