# Supplementary material for: Diversity and recombination analysis of Cotton leaf curl Multan virus: a highly emerging begomovirus in northern India
Source: BMC Genomics. 2019 Apr 6;20:274. doi: 10.1186/s12864-019-5640-2 (PMC6451280; doi:10.1186/s12864-019-5640-2)
Supplement: Supplementary file 1 — Figure S1. PCR based detection of coat protein gene of begomovirus associated with cotton leaf curl disease (BAC) in symptomatic leaf samples using primers specific for the CP gene of BAC. (DOC 324 kb) [file 12864_2019_5640_MOESM1_ESM.doc]

**Diversity and Recombination analysis of *Cotton leaf curl Multan virus*: a highly emerging begomovirus in northern India.**

**Authors**: Razia Qadir, Zainul A. Khan, Dilip Monga, Jawaid A. Khan*

*Plant Virus Laboratory, Department of Biosciences, Jamia Millia Islamia, New Delhi 110025, India. Email: [jkhan1@jmi.ac.in](mailto:jkhan1@jmi.ac.in)

Additional file 1: **Fig. S1**. PCR based detection of coat protein gene of begomovirus associated with cotton leaf curl disease (BAC) in symptomatic leaf samples using primers specific for the CP gene of BAC.


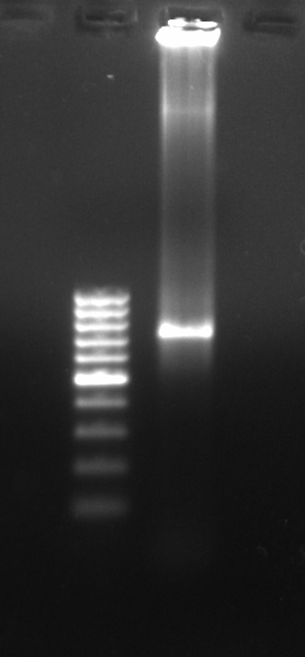


**1 2**

**750 bp**

**500 bp**

Lane 1: molecular size marker (100 bp DNA ladder)

Lane 2: PCR amplification of coat protein gene of Cotton leaf curl virus
