# Supplementary material for: Diversity and recombination analysis of Cotton leaf curl Multan virus: a highly emerging begomovirus in northern India
Source: BMC Genomics. 2019 Apr 6;20:274. doi: 10.1186/s12864-019-5640-2 (PMC6451280; doi:10.1186/s12864-019-5640-2)
Supplement: Supplementary file 2 — Table S1. Nucleotide positions and coding capacity of predicted genes of isolates of Cotton leaf curl Multan virus. (DOC 36 kb) [file 12864_2019_5640_MOESM2_ESM.doc]

**Diversity and Recombination analysis of *Cotton leaf curl Multan virus*: a highly emerging begomovirus in northern India.**

**Authors**: Razia Qadir, Zainul A. Khan, Dilip Monga, Jawaid A. Khan*

*Plant Virus Laboratory, Department of Biosciences, Jamia Millia Islamia, New Delhi 110025, India. Email: [jkhan1@jmi.ac.in](mailto:jkhan1@jmi.ac.in)

Additional file 2: **Table S1.** Nucleotide positions and coding capacity of predicted genes of isolates of *Cotton leaf curl Multan virus*.

| Begomoviruses associated with cotton leaf curl disease | | | | | | | | | | | | | | |
| --- | --- | --- | --- | --- | --- | --- | --- | --- | --- | --- | --- | --- | --- | --- |
| Accession number  &  Size in nucleotides  (This study) | CP (V1) | | V2 | | Rep (C1) | | TrAP (C2) | | REn (C3) | | C4 | | C5 | |
|  | Position (nts) | Coding capacity (no. of amino acids/  kDa) | Position (nts) | Coding capacity (no. of amino acids/ kDa) | Position (nts) | Coding capacity (no. of amino acids/ kDa) | Position (nts) | Coding capacity (no. of amino acids/ kDa) | Position (nts) | Coding capacity (no. of amino acids/ kDa) | Position (nts) | Coding capacity  (no. of amino acids/ kDa) | Position (nts) | Coding capacity  (no. of amino acids/ kDa) |
| SR13; KJ868820 (2757) | 284-1054 | 256 (29.7) | 121-489 | 122 (14.4) | 1503-2594 | 363 (40.9) | 1154-1606 | 150 (17.6) | 1057-1461 | 134 (15.6) | 2135-2437 | 100  (11.2) |  |  |
| SR14; KX951460 (2748) | 278-1048 | 256 (29.7) | 118-474 | 118 (13.85) | 1497-2585 | 362 (40.8) | 1148-1600 | 150 (17.3) | 1051-1455 | 134 (15.6) | 2129-2431 | 100 (11.04) | 62-793 | 243 (27.51) |
| ND14; KX951461  (2751) | 278-1048 | 256 (29.7) | 118-483 | 121  (14.2) | 1497-2588 | 363 (40.9) | 1148-1600 | 150  (17.3) | 1051-1455 | 134 (15.6) | 2129-2431 | 100 (11.06) |  |  |
| SR15; KY888163  (2751) | 278-1048 | 256 (29.7) | 118-474 | 118 (13.85) | 1497-2585 | 362 (40.8) | 1148-1600 | 150 (17.3) | 1051-1455 | 134 (15.6) | 2129-2431 | 100  (11.1) | 62-793 | 243  (27.51) |
| ND15; KY561820  (2750) | 278-1048 | 256 (29.7) | 118-483 | 121  (14.2) | 1497-2588 | 363 (41.0) | 1148-1600 | 150  (17.3) | 1051-1455 | 134 (15.6) | 2129-2431 | 100 (11.1) |  |  |
