# Supplementary material for: Diversity and recombination analysis of Cotton leaf curl Multan virus: a highly emerging begomovirus in northern India
Source: BMC Genomics. 2019 Apr 6;20:274. doi: 10.1186/s12864-019-5640-2 (PMC6451280; doi:10.1186/s12864-019-5640-2)
Supplement: Supplementary file 4 — Table S2. Position and coding capacity of predicted gene of Cotton leaf curl Multan betasatellite molecules associated with cotton leaf curl diseased cotton plants. (DOC 33 kb) [file 12864_2019_5640_MOESM4_ESM.doc]

**Diversity and Recombination analysis of *Cotton leaf curl Multan virus*: a highly emerging begomovirus in northern India.**

**Authors**: Razia Qadir, Zainul A. Khan, Dilip Monga, Jawaid A. Khan*

*Plant Virus Laboratory, Department of Biosciences, Jamia Millia Islamia, New Delhi 110025, India. Email: [jkhan1@jmi.ac.in](mailto:jkhan1@jmi.ac.in)

Additional file 4: **Table S2.** Position and coding capacity of predicted gene of *Cotton leaf curl Multan betasatellite* molecules associated with cotton leaf curl diseased cotton plants.

| Accession number  & Size in nucleotides  (this study) | *Cotton leaf curl Multan betasatellite* | |
| --- | --- | --- |
|  | β*C1* | |
|  | Position (nucleotide) | Coding capacity (no. of amino acids/ kDa) |
| CLCuMB-SR13; KJ868821  (1347) | 200-562 | 120 (13.8) |
| CLCuMB-SR14; KX951462  (1362) | 195-551 | 118 (13.8) |
| CLCuMB-ND14; KX966003  (1371) | 195-551 | 118 (13.9) |
| CLCuMB-ND15; KY817991  (1371) | 195-551 | 118 (13.8) |
