# Supplementary material for: Diversity and recombination analysis of Cotton leaf curl Multan virus: a highly emerging begomovirus in northern India
Source: BMC Genomics. 2019 Apr 6;20:274. doi: 10.1186/s12864-019-5640-2 (PMC6451280; doi:10.1186/s12864-019-5640-2)
Supplement: Supplementary file 5 — Table S3. Position and coding capacity of predicted gene for Guar leaf curl alphasatellite molecules associated with cotton leaf curl diseased cotton plants. (DOC 32 kb) [file 12864_2019_5640_MOESM5_ESM.doc]

**Diversity and Recombination analysis of *Cotton leaf curl Multan virus*: a highly emerging begomovirus in northern India.**

**Authors**: Razia Qadir, Zainul A. Khan, Dilip Monga, Jawaid A. Khan*

*Plant Virus Laboratory, Department of Biosciences, Jamia Millia Islamia, New Delhi 110025, India. Email: [jkhan1@jmi.ac.in](mailto:jkhan1@jmi.ac.in)

Additional file 5: **Table S3.** Position and coding capacity of predicted gene for *Guar leaf curl alphasatellite* molecules associated with cotton leaf curl diseased cotton plants.

| Accession number  & Size in nucleotides  (this study) | *Guar leaf curl alphasatellite* | |
| --- | --- | --- |
|  | Alpha Rep | |
|  | Position (nucleotide) | Coding capacity (no. of amino acids/ kDa) |
| GLCuA-SR14; KX987149  (1368) | 184-1032 | 282 |
| GLCuA-SR15; KY848800  (1361) | 184-1032 | 282 (32.5) |
| GLCuA-ND14; KX987150  (1368) | 82-1029 | 315 (36.4) |
