# Supplementary material for: Diversity and recombination analysis of Cotton leaf curl Multan virus: a highly emerging begomovirus in northern India
Source: BMC Genomics. 2019 Apr 6;20:274. doi: 10.1186/s12864-019-5640-2 (PMC6451280; doi:10.1186/s12864-019-5640-2)
Supplement: Supplementary file 6 — Figure S3. (a-d) Sequence Demarcation Tool based pairwise sequence comparisons. Colour-coded pairwise identity matrix generated from betasatellite genomes. Each coloured cell represents a percentage identity score between two sequences (one indicated horizontally to the left and the other vertically at the bottom). Sequences identified in this study are indicated in the red box. (DOC 718 kb) [file 12864_2019_5640_MOESM6_ESM.doc]

**Diversity and Recombination analysis of *Cotton leaf curl Multan virus*: a highly emerging begomovirus in northern India.**

**Authors**: Razia Qadir, Zainul A. Khan, Dilip Monga, Jawaid A. Khan*

*Plant Virus Laboratory, Department of Biosciences, Jamia Millia Islamia, New Delhi 110025, India. Email: [jkhan1@jmi.ac.in](mailto:jkhan1@jmi.ac.in)

Additional file 6: **Fig. S3 (a-d).** Sequence Demarcation Tool based pairwise sequence comparisons. Colour-coded pairwise identity matrix generated from betasatellite genomes. Each coloured cell represents a percentage identity score between two sequences (one indicated horizontally to the left and the other vertically at the bottom). Sequences identified in this study are indicated in the red box.


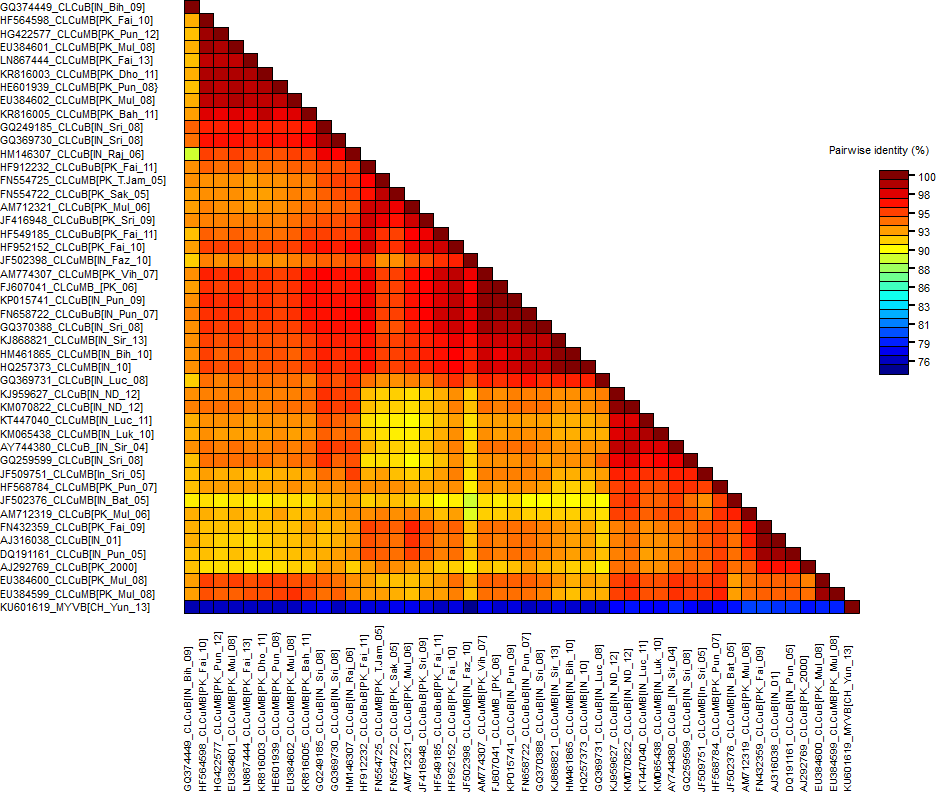


**a)**


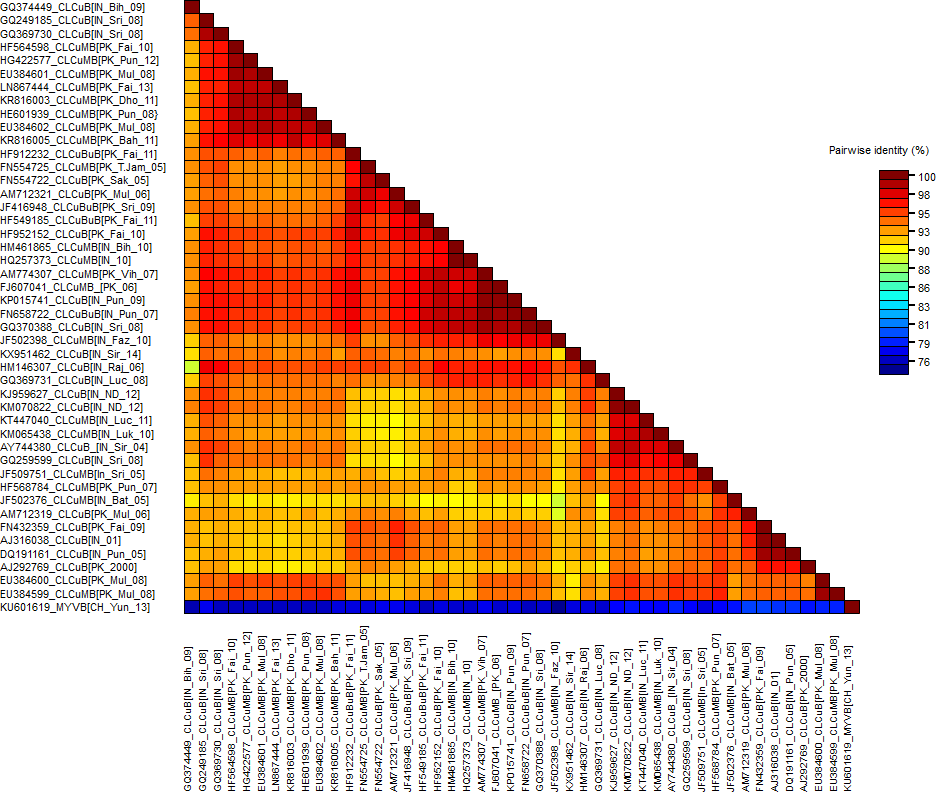


**b)**


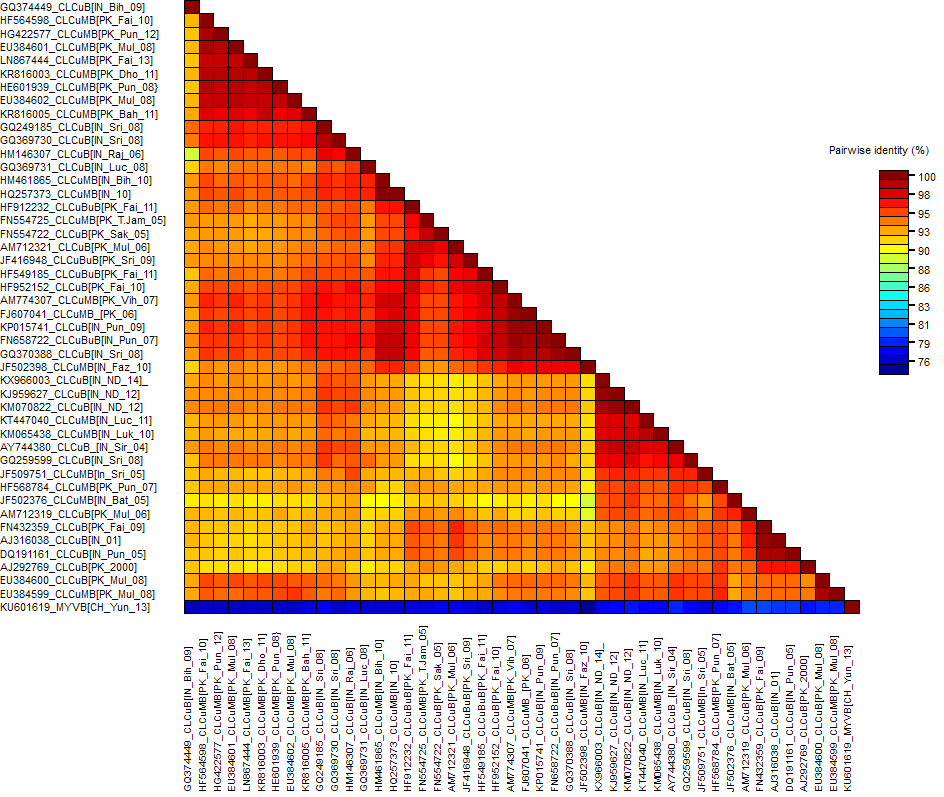


**c)**


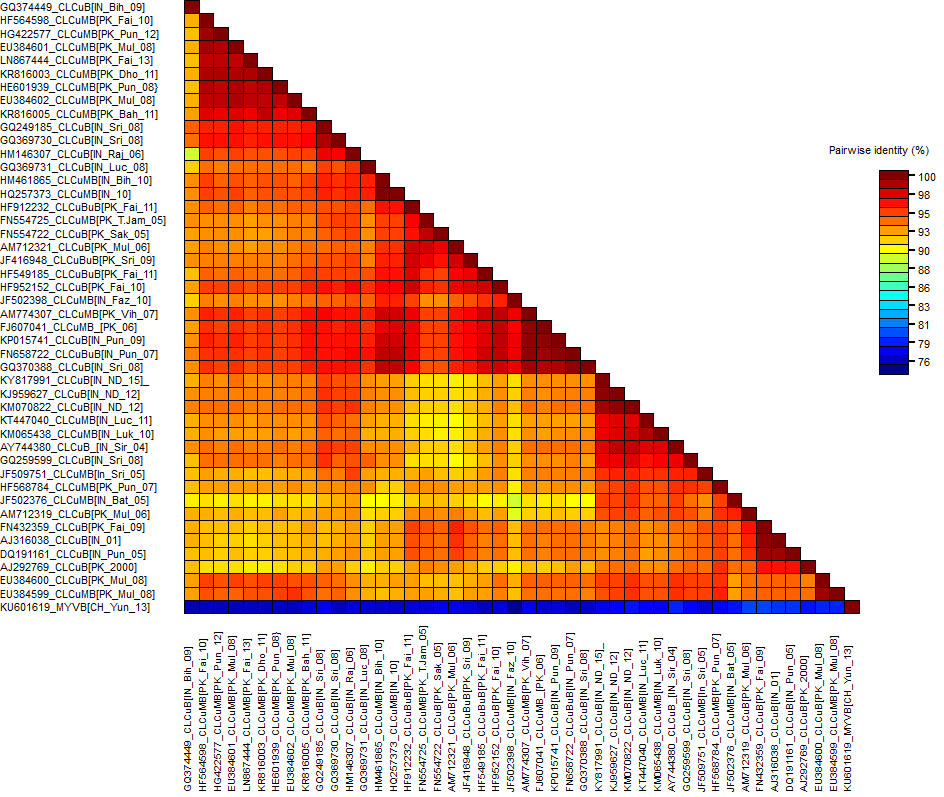


**d)**
