# Supplementary material for: Diversity and recombination analysis of Cotton leaf curl Multan virus: a highly emerging begomovirus in northern India
Source: BMC Genomics. 2019 Apr 6;20:274. doi: 10.1186/s12864-019-5640-2 (PMC6451280; doi:10.1186/s12864-019-5640-2)
Supplement: Supplementary file 8 — Table S4. List of nucleotide sequences of begomoviruses assocated with CLCuD used for the phylogenetic and SDT analysis. (DOC 73 kb) [file 12864_2019_5640_MOESM8_ESM.doc]

**Diversity and Recombination analysis of *Cotton leaf curl Multan virus*: a highly emerging begomovirus in northern India.**

**Authors**: Razia Qadir, Zainul A. Khan, Dilip Monga, Jawaid A. Khan*

*Plant Virus Laboratory, Department of Biosciences, Jamia Millia Islamia, New Delhi 110025, India. Email: [jkhan1@jmi.ac.in](mailto:jkhan1@jmi.ac.in)

Additional file 8: **Table S4.** List of nucleotide sequences of begomoviruses assocated with CLCuD used for the phylogenetic and SDT analysis.

| **Accession numbers of begomoviruses** | **Sampling date** | **Country** | **Place** |
| --- | --- | --- | --- |
| KJ868820 | 2012 | India | Sirsa,Haryana |
| JN678804 | 2010 | India | SriGanganagar,Rajasthan |
| EU384573 | 2006 | Pakistan | Multan, Faisalabad |
| AJ496461 | 1992 | Pakistan | Faisalabad |
| AJ002447 | 1995 | Pakistan | Faisalabad |
|  | 1992 | Pakistan | Faisalabad |
| KY561820 | 2015 | India | New Delhi |
| KX951461 | 2014 | India | New Delhi |
| JN678806 | 2010 | India | Sri Ganganagar, Rajasthan |
| GQ924756 | 2010 | China | Nanning |
| EF465535 | 2006 | China | Guangdong |
| KF766949 | 2013 | China | Guangdong |
| KF766945 | 2013 | China | Guangdong |
| JX914662 | 2012 | China | Jiangsu |
| JQ943408 | 2011 | China | Guangdong |
| GQ503175 | 2008 | China | Guangxi |
| JX286664 | 2011 | China | Guangdong |
| JX286658 | 2011 | China | Guangdong |
| JX286656 | 2012 | China | Guangdong |
| KF766951 | 2013 | China | Guangdong |
| JQ424826 | 2011 | China | Guangdong |
| KF444948 | 2012 | China | Hainan |
| KF413616 | 2012 | China | Guangdong |
| JX861210 | 2012 | China | Fujian |
| JX286660 | 2012 | China | Guangdong |
| JQ963629 | 2011 | China | Guangdong |
| JQ317603 | 2011 | China | Guangxi |
| JN968573 | 2007 | China | Guangdong |
| GU574208 | 2009 | China | Guangxi |
| FJ770370 | 2008 | China | Guangdong |
| KY888163 | 2015 | Sirsa | Haryana |
| KC171654 | 2012 | China | Guangdong |
| KX951460 | 2014 | Sirsa | Haryana |
| JQ963625 | 2011 | China | Guangdong |
| AJ132430 | 1997 | Pakistan | Fisalabad |
| AJ002459 | 1996 | Pakistan |  |
| JN678803 | 2010 | India | Sri Ganganagar |
| FJ218486 | 2006 | Pakistan | Faisalabad |
| EU365615 | 2006 | Pakistan | Multan |
| EU384574 | 2006 | Pakistan | Multan |
| HF549182 | 2011 | Pakistan | Layyah |
| EU365614 | 2006 | Pakistan |  |
| FJ218487 | 2006 | Pakistan | Multan |
| AY765257 | 1999 | India | Ludhiana, Punjab |
| JF502358 | 2005 | India | Bathinda |
| AY765254 | 1999 | India | Sirsa, Haryana |
| AY765253 | 1999 | India | Sirsa, Haryana |
| DQ191160 | 2005 | India | Bathinda, Punjab |
| AY765256 | 1999 | India | Delhi |
| JN807763 | 2011 | India | Lucknow |
| JN880418 | 2011 | India | Lucknow |
| EU365613 | 2006 | Pakistan |  |
| JF502359 | 2005 | India | Sri Ganganagar |
| JX679250 | 2010 | China | Chongqing |
