# Supplementary material for: Diversity and recombination analysis of Cotton leaf curl Multan virus: a highly emerging begomovirus in northern India
Source: BMC Genomics. 2019 Apr 6;20:274. doi: 10.1186/s12864-019-5640-2 (PMC6451280; doi:10.1186/s12864-019-5640-2)
Supplement: Supplementary file 9 — Table S5. List of nucleotide sequences of betasatellites used for phylogenetic and SDT analysis. (DOC 67 kb) [file 12864_2019_5640_MOESM9_ESM.doc]

**Diversity and Recombination analysis of *Cotton leaf curl Multan virus*: a highly emerging begomovirus in northern India.**

**Authors**: Razia Qadir, Zainul A. Khan, Dilip Monga, Jawaid A. Khan*

*Plant Virus Laboratory, Department of Biosciences, Jamia Millia Islamia, New Delhi 110025, India. Email: [jkhan1@jmi.ac.in](mailto:jkhan1@jmi.ac.in)

Additional file 9: **Table S5**. List of nucleotide sequences of betasatellites used for phylogenetic and SDT analysis.

| **Accession numbers of betasatellites** | **Sampling date** | **Country** | **Place** |
| --- | --- | --- | --- |
| KJ868821 | 2012 | India | Sirsa, Haryana |
| AM774307 | 2007 | Pakistan | Vehari |
| HM461865 | 2010 | India | Bihar |
| FJ607041 | 2006 | Pakistan |  |
| JF502398 | 2010 | India | Haryana |
| GQ369731 | 2008 | India | Lucknow |
| HQ257373 | 2010 | India | Punjab |
| KX951462 | 2014 | India | Sirsa |
| KX966003 | 2014 | India | New Delhi |
| KY817991 | 2015 | India | New Delhi |
| KJ959627 | 2012 | India | New Delhi |
| KM070822 | 2012 | India | New Delhi |
| AY744380 | 2004 | India | Sirsa |
| KT447040 | 2011 | India | Lucknow |
| GQ259599 | 2008 | India | Sri Ganganagar, Rajasthan |
| GQ249185 | 2008 | India | Sri Ganganagar, Rajasthan |
| HM146307 | 2006 | India | Mohanpura, Rajasthan |
| HF568784 | 2007 | Pakistan | Multan, Punjab |
| JF502376 | 2005 | India | Bathinda |
| AM712319 | 2006 | Pakistan | Multan |
| EU384600 | 2008 | Pakistan | Multan |
| GQ369730 | 2008 | India | SriGanganagar,Rajasthan |
| EU384599 | 2008 | Pakistan | Multan |
| HF564598 | 2010 | Pakistan | Faisalabad |
| LN867444 | 2013 | Pakistan | Faisalabad |
| KR816003 | 2011 | Pakistan | Dhok Ali Khan |
| GQ374449 | 2009 | India | Bihar |
| EU384602 | 2008 | Pakistan | Multan |
| KR816005 | 2011 | Pakistan | Bahawalnagar |
| FN432359 | 2009 | Pakistan | Faisalabad, Samundri |
| AJ316038 | 2001 | India |  |
| AJ292769 | 2000 | Pakistan |  |
| DQ191161 | 2005 | India | Punjab |
| KM065438 | 2010 | India | Lucknow |
| HF912232 | 2011 | Pakistan | Faisalabad |
| FN554725 | 2005 | Pakistan | Tandojam |
| AM712321 | 2006 | Pakistan | Multan |
| JF509751 | 2005 | India | Sri Ganganagar, Rajasthan |
| JF416948 | 2009 | Pakistan | Sri Ganganagar, Rajasthan |
| FN554722 | 2005 | Pakistan | Sakrand |
| HF952152 | 2010 | Pakistan | Faisalabad |
| HE601939 | 2008 | Pakistan | Punjab |
| HG422577 | 2012 | Pakistan | Punjab |
| FN658722 | 2007 | India | Punjab |
| GQ370388 | 2008 | India | Sri Ganganagar, Rajasthan |
| KP015741 | 2009 | India | Punjab |
| HF549185 | 2011 | Pakistan | Faisalabad |
| KU601619 | 2013 | China | Yunnan |
