# Supplementary material for: Diversity and recombination analysis of Cotton leaf curl Multan virus: a highly emerging begomovirus in northern India
Source: BMC Genomics. 2019 Apr 6;20:274. doi: 10.1186/s12864-019-5640-2 (PMC6451280; doi:10.1186/s12864-019-5640-2)
Supplement: Supplementary file 10 — Table S6. List of alphasatellite sequences used for phylogenetic and SDT analysis. (DOC 77 kb) [file 12864_2019_5640_MOESM10_ESM.doc]

**Diversity and Recombination analysis of *Cotton leaf curl Multan virus*: a highly emerging begomovirus in northern India.**

**Authors**: Razia Qadir, Zainul A. Khan, Dilip Monga, Jawaid A. Khan*

*Plant Virus Laboratory, Department of Biosciences, Jamia Millia Islamia, New Delhi 110025, India. Email: [jkhan1@jmi.ac.in](mailto:jkhan1@jmi.ac.in)

Additional file 10: **Table S6.** List of alphasatellite sequences used for phylogenetic and SDT analysis.

| **Accession numbers of alphasatellites** | **Sampling date** | **Country** | **Place** |
| --- | --- | --- | --- |
| KX987149 | 2014 | India | Sirsa, Haryana |
| KX987150 | 2014 | India | New Delhi |
| KY848800 | 2015 | India | Sirsa, Haryana |
| HE599397 | 2010 | Pakistan | Faisalabad |
| HG417075 | 2012 | Pakistan | Rajoa Sadaat |
| HE599396 | 2010 | Pakistan | Faisalabad |
| HG417076 | 2012 | Pakistan | Jamia Abad |
| HG417078 | 2012 | Pakistan | Rajoa Sadaat |
| HG417077 | 2012 | Pakistan | Rajoa Sadaat |
| HG417072 | 2012 | Pakistan | Rajoa Sadaat |
| HG934793 | 2013 | Pakistan | Rajowal |
| HG934794 | 2013 | Pakistan | Rajowal |
| HG934789 | 2013 | Pakistan | Bangaypur |
| JX262389 | 2010 | India | Mohali, Punjab |
| HG417074 | 2012 | Pakistan | Rajoa Sadaat |
| HE979547 | 2011 | Pakistan | Faisalabad |
| KC305096 | 2011 | India | Mohali |
| KC305095 | 2011 | India | Wellington |
| KT390435 | 2014 | India | Ludhiana, Punjab |
| KT390427 | 2013 | India | Haryana, Karnal |
| HQ180392 | 2009 | India | Pusa, Bihar |
| HG515060 | 2012 | Pakistan | Lassa lawaris |
| HG515062 | 2012 | Pakistan | Lassa lawaris |
| LN713499 | 2014 | Pakistan | Sindh |
| LN713491 | 2014 | Pakistan | Sindh |
| LN713489 | 2014 | Pakistan | Sindh |
| LN713490 | 2014 | Pakistan | Sindh |
| HG934825 | 2013 | Pakistan | Adda, Janiwala |
| KY001658 | 2010 | Nepal |  |
| KY001655 | 2010 | Nepal |  |
| KY001659 | 2010 | Nepal |  |
| HG934826 | 2013 | Pakistan | Adda 288, Janiwala |
| LN831729 | 2014 | Pakistan | LCWU, |
| GU385877 | 2010 | India | Lucknow |
| KT390423 | 2013 | India | Karnal, Haryana |
| HG515067 | 2012 | Pakistan | Lassa lawaris |
| HG515061 | 2012 | Pakistan | Lassa lawaris |
| KF471054 | 2008 | India | Varanasi |
| KT390408 | 2013 | India | Varanasi, Uttar Pradesh |
| KT390497 | 2014 | India | Varanasi, Uttar Pradesh |
| KT390414 | 2013 | India | Hyderabad, Andhra Pradesh |
| KT390410 | 2013 | India | Mirzapur, Uttar Pradesh |
| KT390421 | 2013 | India | Karnal, Haryana |
| KT390507 | 2014 | India | Varanasi, Uttar Pradesh |
| HG417071 | 2012 | Pakistan | RajoaSadaat |
| HG417073 | 2012 | Pakistan | RajoaSadaat |
| KT390433 | 2014 | India | Ludhiana, Punjab |
| KF471053 | 2008 | India | Varanasi |
| KF471055 | 2008 | India | New Delhi |
| KT390429 | 2013 | India | Karnal, Haryana |
| KT390409 | 2013 | India | Mirzapur, Uttar Pradesh |
| KC677736 | 2011 | Japan | Tokyo |
| KC282643 | 2010 | Nepal |  |
| KT390504 | 2014 | India | Varanasi, Uttar Pradesh |
| JF733780 | 2009 | China | Zhejiang |
