# Supplementary material for: Diversity and recombination analysis of Cotton leaf curl Multan virus: a highly emerging begomovirus in northern India
Source: BMC Genomics. 2019 Apr 6;20:274. doi: 10.1186/s12864-019-5640-2 (PMC6451280; doi:10.1186/s12864-019-5640-2)
Supplement: Supplementary file 11 — Table S7. List of nucleotide sequences of begomoviruses (a), betasatellites (b) and alphasatellites (c) associated with CLCuD used for recombination analysis. (DOC 39 kb) [file 12864_2019_5640_MOESM11_ESM.doc]

**Diversity and Recombination analysis of *Cotton leaf curl Multan virus*: a highly emerging begomovirus in northern India.**

**Authors**: Razia Qadir, Zainul A. Khan, Dilip Monga, Jawaid A. Khan*

*Plant Virus Laboratory, Department of Biosciences, Jamia Millia Islamia, New Delhi 110025, India. Email: [jkhan1@jmi.ac.in](mailto:jkhan1@jmi.ac.in)

Additional file 11: **Table S7.** List of nucleotide sequences of begomoviruses (a), betasatellites (b) and alphasatellites (c) associated with CLCuD used for recombination analysis.

**a)**

| **BAC isolates (this study)** | **Sequences used for recombination analysis (source: GenBank)** |
| --- | --- |
| CLCuMuV-SR14  (KX951460) | KX951460, JN678804, AJ002447, EU384573, AJ002458, AJ496287, JN678806, DQ191160, AJ496461, AY765256, EU365616 , FN645912, JF509748, AY795606, AM501481, JF509746, EU384574, FM202328, KF766949, GQ924756, KF766945, KF766955, JQ424826, KF413618, JF509747, AM774301, JF416947, FR750321, FR750319, FR750320, FN645932, AM774303, AM774302, AM774300, AM774295, HM461863, AM774296 |
| CLCuMuV-ND14  (KX951461) | KX951461, JN678804, EU384573, AJ002447, AJ002458, HF549182, JN678806, AJ496287, AJ496461, AY765253, DQ191160, AY765256, GQ924758, EF465535, KF766955, KF766949, KF766945, KF413618, JX861210, JQ963631, JX914662, KF766951, KF444948, JX286660, JQ424826, KF413616, JX286662, JQ943408, JN968573, GQ503175, KF766953, KF766947, JX286664, JX286658, JQ963629, JQ963627, JQ317603, FJ770370, JX286656, GU574208, KC171654, JQ963625, AM712436 |
| CLCuMuV-ND15  (KY561820) | KY561820, JN678804, EU384573, AJ002447, AJ002458, JN678806, AJ496287, HF549182, AY765253, KF766955, KF766945, KF766949, KF413618, JQ963631, JX914662, KF766951, KF444948, JX861210, JX286660, JQ424826, KF413616, JX286662, GQ503175, KF766947, JX286664, JX286658, JQ963629, JQ963627, JQ317603, JX286656, GU574208, FJ770370, KC171654, JQ963625, AJ32430, AJ002459, EU365616, HQ158010, AY765257, JF509749, GQ220850, JF509748, AM501481, AY795605, JF509746, AY795606, JN678803, FJ218486, EU365615, JF509747, JF502369, HF549181, AJ8902228 |
| CLCuMuV-SR15  (KY888163) | KY888163, KM096471, KM09646, KJ868820, JN678804, AJ002447, AJ002458, EU384573, AJ496287, KJ959628, JN678806, AJ496461, DQ191160, HF549182, KX831888, KM096470, KM096467, KM096469, KX831891, KJ959630, KT228327, KM065514, HQ158010, FN645912, KJ959629, JF509748, GQ220850, JF509749, AY795606, AM501481, AY795605, KM096468, AF363011, JF509746, AY795607, HG937521, JF502369, FM202328, KC412251, JF502363, HM235774, JF502362, JF502364, JN678803, KR135370, JF502361, HM037920, FN552005, FN552004, FN552001, FN552002, FN552003, JN558352, FJ218487, JF509747, KT390455, HG937519, LN713479, LN811060, HE995543, HF549181, JF502365, LN845933, HE995547, FM164938 |

**b)**

| **Betasatellite isolates (this study)** | **Sequences used for recombination analysis (GenBank)** |
| --- | --- |
| CLCuMB-SR13  (KJ868821) | KJ868821, KJ959627, KM070822, AY744380, KT447040, KM065438, AY795608, JX217745, GQ370389, GQ259599, GQ249185, HM146307, HF568784, JF502376, JF502391,, AM712319, EU384605, EU384604, EU384600, EU384580, HE601938, GQ369730, EU384599, KT228323, HF564598, EU384601, LN867444, EU384598, KR816003, HG4222583, HG422578, HG000665, HE978342, HE601939 |
| CLCuMB-ND14  (KX966003) | KX966003, KJ959627, KM070822, AY744380, KT447040, KM065438, AY795608, JX217745, GQ370389, GQ259599, GQ249185, HM146307, HF568784, JF502376, AM712319, EU384605, EU384604, EU384600, EU384580, HE601938, GQ369730, EU384599, KT228323, HF564598, EU384601, LN867444, EU384598, KR816003, HG4222583, HG422578, HG000665, HE978342, HE601939 |
| CLCuMB-SR14  (KX951462) | KX951462, KT228323, HM146307, GQ249185, KM070822, KJ959627, KT447040, KT228325, KM065438, GQ370389, LK995398, HF912232, FN554725, JF509751, EU862816, GQ369730, JF502384, JF416948, KT228326, FN554723, FN554722, AM712321, JF502392, AM712323, HF952153, HF952152, HE601940, HE601939, HG422577, FN554724, KJ868821, FN658722, HF564599, GQ370388, KP015741, HF549185 |

**c)**

| **Alphasatellite isolates (this study)** | **Sequences used for recombination analysis (GenBank)** |
| --- | --- |
| GLCuA-ND14  (KX987150) | KX987150, HG417075, HG417076, HG417077, HG417078, HE599396, HG417072, HG417071, HE599397, HG417073, HG417074, HE979547, KC305096, KC305095, KT390435, KT390427, GU385877, KT390423, HQ180392, HG515067, HG515061, HG515060, HG515062, KF471053, KF471054, KF471055, KT390429, KT390408, KT390497, KT390414, KT390410, KT390409, HG518790, KC677736, KC282643, KT390504, KT390421, KT390507, JF733780 |
